# Single‐Molecule Characterization and Super‐Resolution Imaging of Alzheimer's Disease‐Relevant Tau Aggregates in Human Samples

**DOI:** 10.1002/anie.202317756

**Published:** 2024-04-17

**Authors:** Dorothea Böken, Dezerae Cox, Melanie Burke, Jeff Y. L. Lam, Taxiarchis Katsinelos, John S. H. Danial, Emre Fertan, William A. McEwan, James B. Rowe, David Klenerman

**Affiliations:** ^1^ Yusuf Hamied Department of Chemistry University of Cambridge Cambridge CB2 1EW UK; ^2^ UK Dementia Research Institute University of Cambridge Cambridge CB2 0AH UK; ^3^ MRC Laboratory of Molecular Biology Cambridge CB2 0QH UK; ^4^ Department of Clinical Neurosciences and Cambridge University Hospitals NHS Trust University of Cambridge Cambridge CB2 0SZ UK

**Keywords:** single-molecule studies, proteins, protein aggregation, fluorescence microscopy, neurodegenerative disease

## Abstract

Hyperphosphorylation and aggregation of the protein tau play key roles in the development of Alzheimer's disease (AD). While the molecular structure of the filamentous tau aggregates has been determined to atomic resolution, there is far less information available about the smaller, soluble aggregates, which are believed to be more toxic. Traditional techniques are limited to bulk measures and struggle to identify individual aggregates in complex biological samples. To address this, we developed a novel single‐molecule pull‐down‐based assay (MAPTau) to detect and characterize individual tau aggregates in AD and control post‐mortem brain and biofluids. Using MAPTau, we report the quantity, as well as the size and circularity of tau aggregates measured using super‐resolution microscopy, revealing AD‐specific differences in tau aggregate morphology. By adapting MAPTau to detect multiple phosphorylation markers in individual aggregates using two‐color coincidence detection, we derived compositional profiles of the individual aggregates. We find an AD‐specific phosphorylation profile of tau aggregates with more than 80 % containing multiple phosphorylations, compared to 5 % in age‐matched non‐AD controls. Our results show that MAPTau is able to identify disease‐specific subpopulations of tau aggregates phosphorylated at different sites, that are invisible to other methods and enable the study of disease mechanisms and diagnosis.

## Graphical abstract

Introducing MAPTau, a novel single‐molecule approach for detailed analysis of tau aggregates in human brain and blood, offering unprecedented insights into the quantity, size, and phosphorylation profile of individual tau aggregates. By identifying distinctive Alzheimer's disease‐specific tau aggregate morphologies and phosphorylation patterns, MAPTau opens new avenues for investigating disease mechanisms and novel diagnostic strategies.

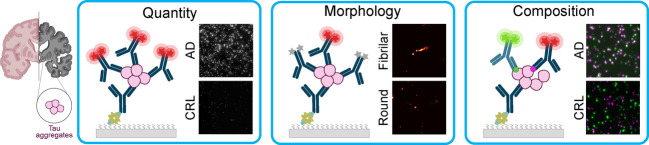

## Introduction

Hyperphosphorylation and aggregation of tau is a defining characteristic of a range of neurodegenerative diseases collectively known as tauopathies, including Alzheimer's disease (AD), progressive supranuclear palsy, and multiple forms of frontotemporal dementia, including Pick's disease.[^1^, ^2^, ^3^, ^4^, ^5^] Tau is an essential protein involved in the stabilization of microtubules.[^6^, ^7^] However, hyperphosphorylation of tau causes it to dissociate from microtubules leading to its aggregation.^[8]^ While the exact cause of hyperphosphorylation remains unknown, the formation of tau aggregates precedes and potentially causes cognitive decline.^[9]^ Large, insoluble deposits in the form of neurofibrillary tangles (NFTs) containing tau filaments, the product of aggregation, are a primary histopathological hallmark of tauopathies. Indeed, in AD cognitive decline correlates more closely with the progression of tau aggregate pathology than with the presence of amyloid‐β plaques.[^10^, ^11^, ^12^] However, small soluble aggregates are thought to exert potent cellular toxicity[^13^, ^14^] and appear to spread from cell to cell as a potential mechanism for the propagation of tau pathology throughout the brain.[^15^, ^16^]

The structure of the ordered part of tau filaments formed in several tauopathies has recently been determined using Cryo‐EM.[^17^, ^18^] Furthermore, very sensitive tau seeding assays have been developed to detect the number of seed competent tau aggregates in postmortem brain samples and CSF.[^19^, ^20^, ^21^] However, despite their importance, the small, soluble tau aggregates that form during the aggregation process are more challenging to study, since they are highly heterogeneous in size, shape, and phosphorylation state.^[22]^

Here, we present a new method to study tau aggregates, Molecular Analysis and Profiling of Tau aggregates (MAPTau). Using an adaptation of the single‐molecule pull‐down (SiMPull) method, MAPTau combines antibody‐based immunoprecipitation with single‐molecule, super‐resolution fluorescence imaging and two‐color coincidence detection.^[23]^ Using MAPTau, it is possible to detect individual aggregates and characterize them in terms of size and shape, enabling the identification of subpopulations, which are potentially linked to disease mechanisms. This is not possible with bulk techniques such as enzyme‐linked immunosorbent assays (ELISA), which quantify the monomer, soluble aggregate, and fibrillar forms of tau together and cannot provide morphological data. In this work we show that MAPTau can quantify and characterize tau aggregates with high specificity and sensitivity in disease‐derived samples, including brain tissue homogenates and serum extracts, revealing differences in tau aggregate number between tauopathy disease‐derived and age‐matched control samples. Finally, using co‐localization and super‐resolution microscopy to characterize aggregate size, shape, and composition we identify disease‐associated aggregate subpopulations. Together, these characteristics confirm MAPTau as a less‐invasive diagnostic tool which can distinguish aggregate subpopulations to track disease pathology and progression in diverse biofluids.

## Results and Discussion

### Establishing MAPTau for the Selective Detection of Tau Aggregates

**Figure 1:**
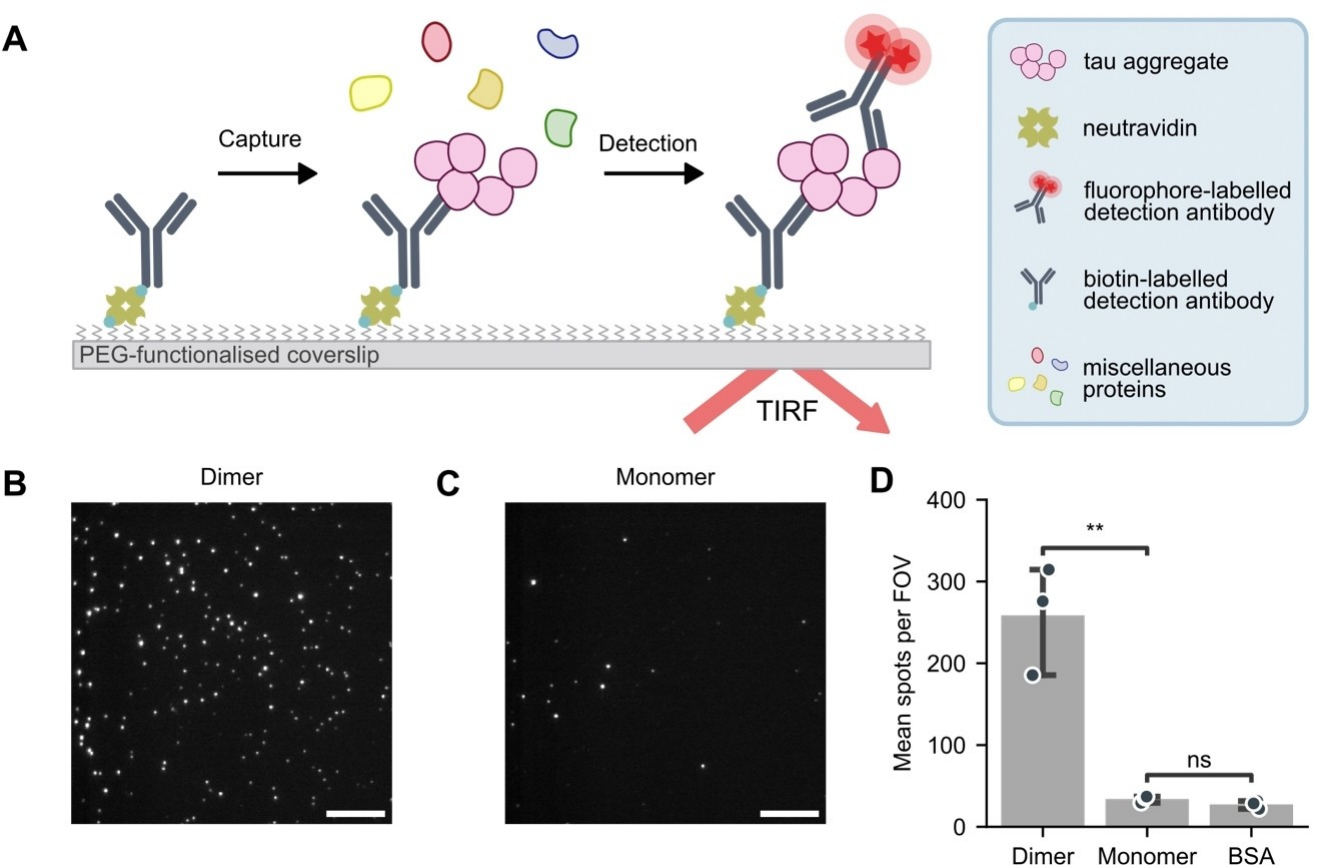
MAPTau aggregate assay specifically detects multimeric particles. (A) Schematic representation of the single‐molecule pull‐down (SiMPull) assay. For the detection of aggregates, the same antibody is used for capture and detection. Images are acquired using total internal reflection fluorescence (TIRF) microscopy. (B) Representative image of MAPTau applied to a dimer‐mimicking peptide, containing two linked HT7 epitope sequences. Scale bar=10 μm. (C) Representative image for MAPTau of a monomer‐mimicking peptide, containing one HT7 epitope and a randomized sequence. (D) Diffraction‐limited quantification of the number of spots in individual fields of view. Panel D shows mean±S.D. of *n*=3 technical replicates, compared using a one‐way Anova with post‐hoc Tukey HSD test. ns: *p*>0.05, **: *p*<0.01.

The single‐molecule pull‐down (SiMPull) assay is an antibody‐based technique that uses fluorescence microscopy to detect and characterize single molecules. Antibodies immobilized on a polyethylene glycol (PEG)‐passivated surface are used to capture tau, which subsequently can be detected using a fluorescently labeled antibody using total internal reflection (TIR) illumination (Figure 1A). For the specific detection of tau aggregates—and not monomers, MAPTau was developed using matched monoclonal antibodies for both capture and detection.^[24]^

We reasoned that this configuration required the presence of two identical epitopes within a single aggregate, as capturing the aggregate would occupy one binding site (epitope) and require a second site for binding of the detection antibody. This ensures that the detected tau species are, at a minimum, dimeric. Further, it allows multimeric tau to be isolated without relying on conformation‐specific antibodies, thereby remaining agnostic to the structure of the tau aggregates. We initially selected two tau antibodies that are well characterized and validated: the phospho‐tau specific antibody AT8 (p‐Ser202, p‐Thr205) and the total tau antibody HT7.^[25]^

We first sought to confirm the specificity of the assay for tau multimers over monomers. We created synthetic peptides comprising two 10‐residue sequences joined with a PEG linker. One peptide contained a single copy of the HT7 epitope tethered to a randomized version of the epitope sequence; this is designated as the ‘monomeric’ peptide given it mimics the presence of the single HT7 binding site available in monomeric tau. The second peptide, designated to mimic the ‘dimeric’ peptide, contained two HT7 epitope sequences tethered together, mimicking the presence of two HT7 binding sites in multimeric tau. Importantly, these peptides maintain comparable physicochemical properties to one another owing to their overall identical composition. As a negative control, we used bovine serum albumin (BSA), a well‐characterized recombinant control protein which does not contain any HT7 binding sites. These samples were compared with MAPTau, where the primary assay readout was the number of fluorescent spots visible in diffraction‐limited images (Figure 1B,C and Supplementary Figure S1A).

As expected, the number of fluorescent spots (signal) was positively correlated with the presence and concentration of the HT7 epitope (Figure 1B and C). Even at very high sample concentrations (0.2 mg/mL), we detected significantly fewer spots in the presence of the monomer‐mimicking peptide (34±4 spots) per field of view (FOV) compared to the dimer mimic (260±66 spots per FOV; Figure 1D; one‐way Anova with post‐hoc Tukey HSD test, *p*=0.0084). In contrast, there was no significant difference in the number of spots between the monomer‐mimicking peptide and the BSA negative control (*p*=0.29). The HT7 dimer‐mimicking peptide was not detected by an antibody targeting a different tau epitope (AT8; Supplementary Figure S1B). These data confirmed the assay is able to distinguish tau multimers from monomers.

We further confirmed the specificity of MAPTau by comparing the detection of (phosphorylated) tau aggregates against other relevant recombinant protein aggregates, namely beta‐amyloid (Aβ) and alpha synuclein (αSyn) aggregates produced in vitro (Supplementary Figure S1C and D). We found on average 12±4 spots per FOV when performing MAPTau on these control samples, which is equivalent to the buffer control without any protein present—there was no significant difference between PBS and amyloid‐β or α‐synuclein (AT8 assay: one‐way ANOVA with post‐hoc Tukey HSD test, *p*=1.0 for PBS versus Aβ, *p*=1.0 for PBS vs α‐synuclein; HT7 assay: one‐way ANOVA *p*=0.0000050; post‐hoc Tukey HSD test, *p*=0.98 for PBS vs amyloid‐β, *p*=1.0 for PBS vs αSyn). Further, we ensured that the recombinant aggregates can be detected with their appropriate antibody combination using corresponding MAPTau assays (Supplementary Figure S1E and F). Taken together, these results confirmed that MAPTau can be used to specifically detect tau multimers.

### Diffraction‐Limited MAPTau Detects Alzheimer's Disease‐Associated Tau Aggregates

**Figure 2:**
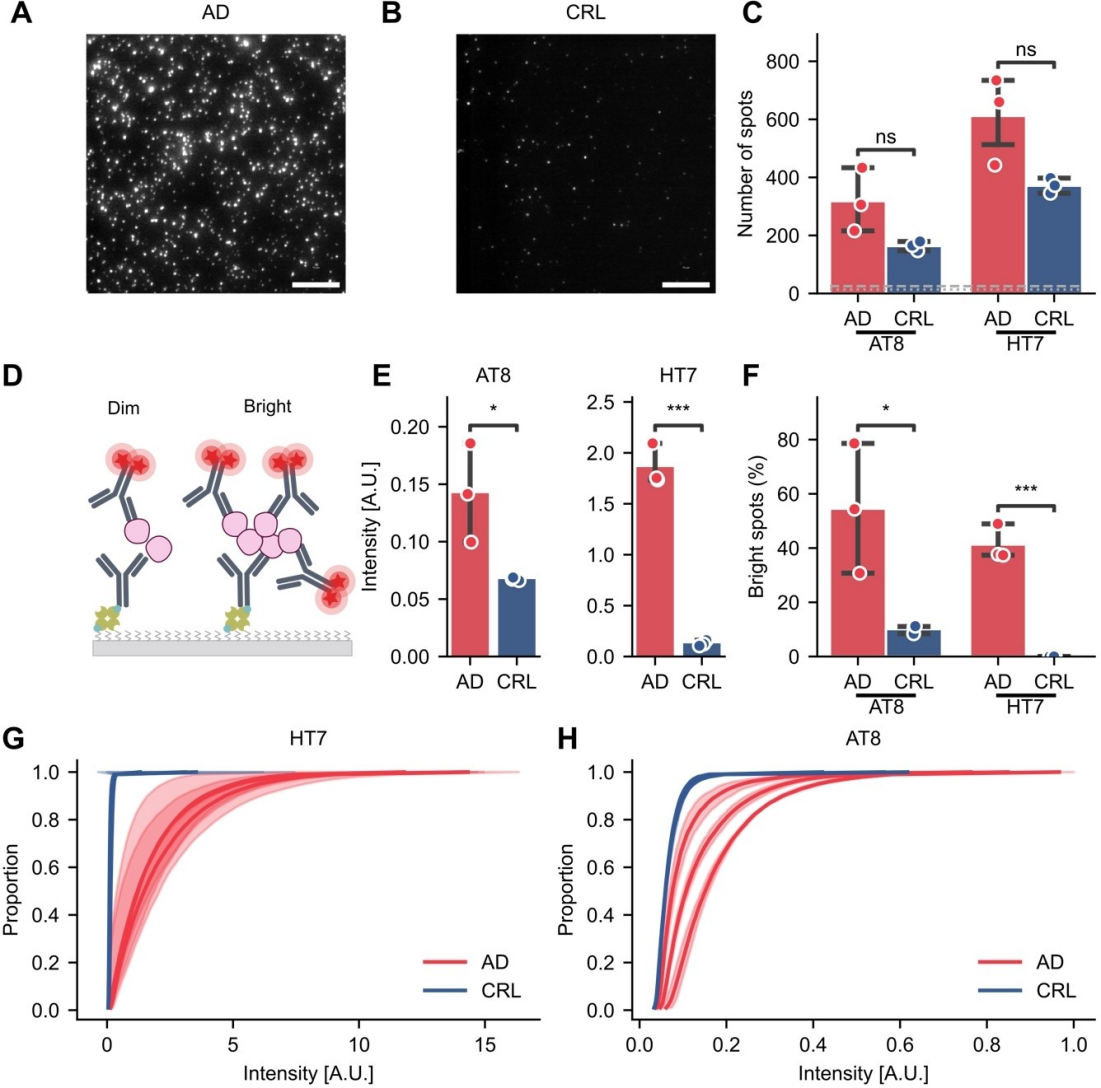
Quantification and characterization of tau aggregates in human brain tissue. (A) Representative image obtained for an AD‐derived sample using AT8. Scale bar=10 μm. (B) Representative image obtained from a control sample using AT8. (C) Quantification of total tau (HT7) and p‐tau (AT8) aggregates from AD (Braak stage VI, *n*=3) and age‐matched control patients (*n*=3) Levels of BSA background are indicated as dotted (AT8) or dashed (HT7) lines. (D) Schematic of detection antibody binding correlated with aggregate size. Mean intensity of aggregates relates to the number of detection antibodies bound and thereby the aggregate size. (E) Mean intensity of tau aggregates in AD, CRL brain and BSA. (F) Percentage of very bright aggregates (AT8: intensity>0.1 A.U., HT7: intensity>1.5 A.U,). (G–H) Cumulative distribution of the aggregate brightness using (G) HT7 or (H) AT8 of *n*=3 biological replicates, showing the S.D. of three technical replicates (shade). Panel C, E, F show the mean±S.D. of *n*=3 biological replicates and asterisks refer to t‐tests: ns: *p*>0.05, *: *p*<0.05, **: *p*<0.01, ***: *p*<0.001.

We proceeded to test a biologically relevant, complex sample, containing a high concentration of tau aggregates. Thus, homogenized human post‐mortem brain tissue samples from donors with clinical and neuropathological evidence of the Alzheimer's tauopathy and age‐matched control donors without AD were analyzed using MAPTau. We confirmed the samples contain tau (not aggregate specific) using a commercial ELISA (Supplementary Figure S1G). We detected on average higher levels of tau in the control brain samples compared to AD (t‐test, *p*=0.11). We then quantified the tau aggregate content in the brain homogenate samples using the HT7 and AT8 MAPTau assays (Figure 2A and B). On average, we detected 600±150 HT7‐positive spots and 320±110 AT8‐positive spots per FOV respectively (Figure 2C). We determined the sensitivity of MAPTau; the limit of detection for the HT7 and AT8 assays was calculated as 874 pg/mL and 2201 pg/ml of tau, respectively^[26]^ (Supplementary Figure S1H). Importantly, since we used a non‐aggregate specific ELISA to determine the tau concentration, this limit is with respect to total (monomeric) tau. We anticipate aggregated tau to comprise only a fraction of the total tau present, and not all tau to be AT8 phosphorylated, meaning the limit of detection for tau aggregates can be expected to be several folds lower in both assay configurations.

There were 1.5x more tau‐positive aggregates in brain homogenate from the frontal cortex of AD patients than age‐matched donor controls (*n*=3) but this was not statistically significant (t‐test, HT7: *p*=0.054, AT8: *p*=0.072). However, when we normalized the number of tau aggregates to the total tau monomer present (detected by ELISA), to obtain the proportion of aggregated tau, there was a significant difference between AD and control samples for HT7 but not AT8 (Supplementary Figure S1I, t‐test, HT7: *p*=0.031, AT8: *p*=0.065). In agreement with previous studies, there was no significant difference in the levels of total tau in AD brain compared to control (t‐test, p=0.096).^[27]^ The levels of HT7‐positive tau aggregates were also higher than the AT8‐positive tau aggregates, which may indicate that not all tau aggregates are AT8 positive or have accessible AT8 epitopes. Interestingly, we also observed aggregate levels above background in the age‐matched control brains, indicating the presence of small tau aggregates even at Braak stage 0/1 (when there are no tangles present in the frontal cortex^[28]^). Given the stark differences between insoluble aggregate accumulation in healthy aging and disease on which Braak staging is predicated, this was unexpected. However, MAPTau can detect small, soluble aggregates which are below the resolution and detection limit of most techniques. The comparable abundance of tau aggregates in AD and control brains suggests differences in aggregates driving disease must lie beyond their abundance. To test this, we morphologically characterized the tau aggregates.

An advantage of MAPTau is its ability to obtain information on each aggregate individually, allowing the measurement of aggregate size and shape, which has been linked to distinct disease mechanisms for other pathological aggregates such as Aβ and ɑSyn.[^24^, ^29^] Specifically, there is additional information in the mean brightness of each spot since this should correlate with aggregate size. Larger aggregates present more epitopes to which fluorophore‐labelled antibodies can bind and hence will be brighter (Figure 2D). The mean brightness of tau aggregates detected in each sample using HT7 or AT8 (Figure 2E) revealed significant differences between the disease and control cohorts (t‐test, HT7: *p*=0.00012, AT8: *p*=0.039). A significantly higher percentage of bright spots (AT8: >0.1 A.U., HT7: >1.5 A.U.) was also observed in the AD samples compared to the control cohort for both antibody configurations (Figure 2F, t‐test, HT7: *p*=0.00041, AT8: *p*=0.033). This is also evident in the cumulative distribution of spot brightness, a comparison that is made possible only by the single‐molecule nature of MAPTau. The distribution of spot brightness′ was highly skewed toward dim (~small) aggregates in control samples, a feature which was consistent across different control donors in both assays (Figure 2G and H). In contrast, AD aggregate profiles were more variable between donors, though consistently showed a greater range in brightness of the aggregates, suggesting a greater diversity of aggregate sizes is present in these samples (Figure 2G and H). Overall, these data support the ability of MAPTau to distinguish between disease‐derived and control aggregate‐containing samples according to tau aggregate abundance and brightness. We detected brighter spots in the AD samples compared to control, suggesting the presence of larger aggregates.

### Super‐Resolved MAPTau Quantifies Aggregate Morphology with Single Aggregate Precision

**Figure 3:**
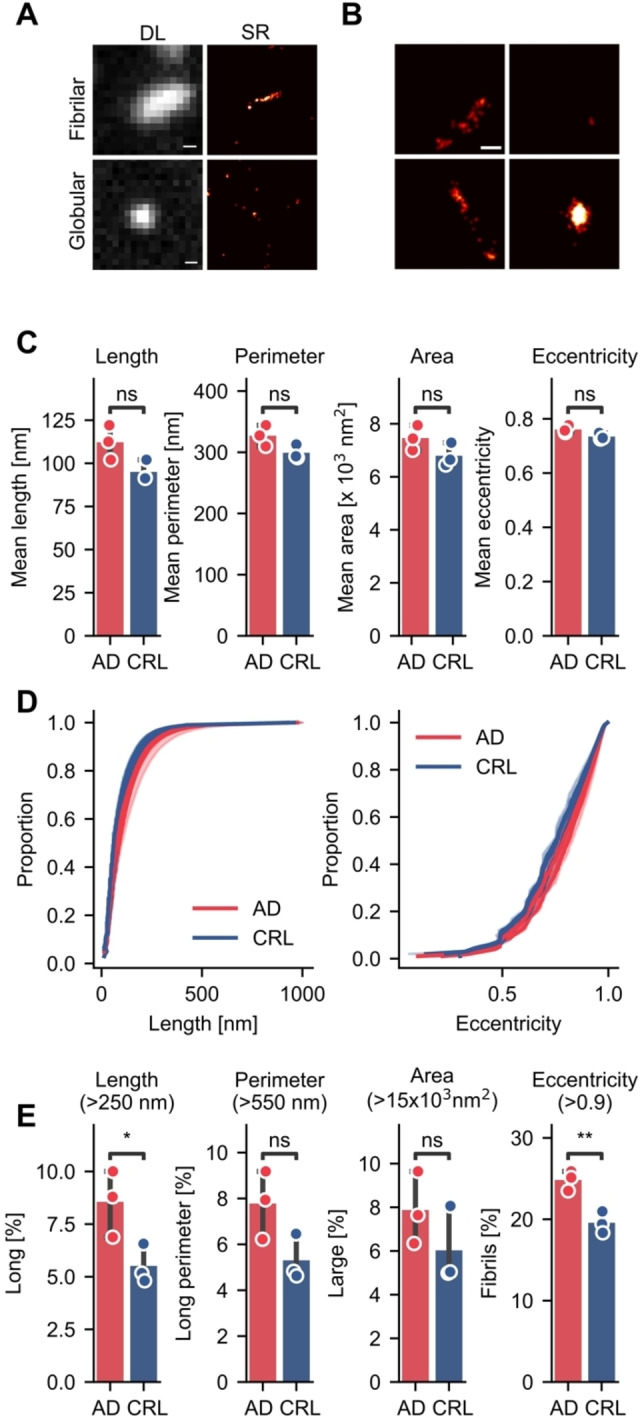
Characterizations of brain‐derived tau aggregates using MAPTau combined with super‐resolution microscopy. (A) Representative images of diffraction‐limited and super‐resolved tau aggregates revealing distinct morphological categories. Scale bar=200 nm. (B) Example images of aggregates of varying size and eccentricity. Scale bar=100 nm. (C) Mean length, perimeter, area, and eccentricity of p‐tau aggregates detected via AT8 MAPTau. (D) Cumulative distribution of aggregate length and eccentricity measured as in C of *n*=3 biological replicates, showing the S.D. of three technical replicates (shade). (E) Proportion of aggregates above the stated thresholds for size (length, perimeter, area) and shape (eccentricity) for brain‐derived samples taken from AD (red) and control (blue) cohorts. Panel C and E show the mean±S.D. of *n*=3 biological replicates using a t‐test. ns: *p*>0.05, *: *p*<0.05, **: *p*<0.01.

To characterize aggregate morphology with a precision unattainable via diffraction‐limited imaging, we adapted MAPTau for super‐resolution microscopy, specifically Stochastic Optical Reconstruction Microscopy (STORM),^[30]^ which has previously been used to image intracellular tau aggregates.[^31^, ^32^] Combining MAPTau with STORM, we were able to quantify the length, perimeter, total area, and eccentricity of individual tau aggregates in brain homogenate. We observed two dominant aggregate shapes, loosely falling into extended ‘fibrillar’ and short ‘globular’ categories (Figure 3A and B and Supplementary Figure S2A). As anticipated, aggregates were overall highly heterogeneous, ranging from 30 nm to more than 400 nm in length. On average, tau aggregates detected in AD brain were 115 nm long while the ones detected in control brains were 95 nm long (*p*=0.064). While we did not observe any significant differences in the mean size or eccentricity shape of the aggregates (Figure 3C), we did observe qualitative separation of the cumulative distribution of aggregate length and eccentricity between disease‐derived and control samples, as the AD samples had a higher proportion of very long aggregates (Figure 3D). This prompted us to consider the proportion of long aggregates (>250 nm), which was on average 1.5× higher in the AD samples compared to control (Figure 3E, t‐test *p*=0.045). Similarly, the proportion of fibrillar aggregates (eccentricity>0.9) was 25 % in AD while only 20 % in the control brains (Figure 3E, t‐test *p*=0.0072). The accumulation of this sub‐population of tau aggregates with specific sizes and shapes is correlated with disease pathology and hence likely to be disease relevant. These findings suggest that normal neurons produce tau aggregates of a range of sizes. However, provided these aggregates are removed as fast as they form, they do not accumulate. In contrast, in AD if there is an imbalance in the production and removal rate in some neurons, then longer, more fibrillar aggregates accumulate leading to an increase in these sub‐populations and ultimately may result in neuronal cell death. The difference between AD samples and controls is small, probably due to dead neurons releasing their cellular contents and hence not contributing to the tau aggregates observed in these experiments. These findings also highlight the necessity of single‐molecule techniques to be able to identify tau aggregate subpopulations, not accessible in population averages.

The combination of size and shape for individual aggregates provides another metric for comparing aggregate populations between cohorts (Supplementary Figure S2B and C). In AD and control samples, longer aggregates (>250 nm) were significantly more likely to be fibrillar; 55 % of long aggregates had an eccentricity>0.9, compared to 5 % of short (<100 nm) aggregates (Supplementary Figure S2D, AD p=0.000018). Interestingly, the percentage of long aggregates in the population of round aggregates (eccentricity <0.7) was significantly higher in AD than in control (AD: 2.5 %, CRL: 1 %, p=0.047) (Supplementary Figure S2E). This is in agreement with the analysis of the free‐energy landscape for tau aggregation which features two pathways; one leading to ordered fibrils and the other to amorphous phases.^[33]^ Furthermore, spontaneous aggregation of in vitro hyperphosphorylated tau forms round aggregates which were able to induce a robust inflammatory response in human macrophages.^[34]^ Thus, both fibrillar and hyperphosphorylated round tau aggregates may be associated with disease pathology, and MAPTau readily enables their distinction in biological contexts.

Our resolution did not allow eccentricity measures above 0.9 for aggregates shorter than 50 nm meaning smaller fibrillar aggregates could not be identified. To address this, we further analyzed the data by filtering out all aggregates shorter than 50 nm in length. First, we confirmed that the difference in the proportion of long aggregates and fibrils between the brains from AD and control patients remained the same as before (Supplementary Figure S2F). However, the difference in the mean length, eccentricity, and perimeter of aggregates between AD and control samples reached significance, once the small aggregates were removed (Supplementary Figure S2G). This suggests that the variability between patients within the AD cohort is driven by aggregates shorter than 50 nm, such that once these are removed the mean values are more consistent within disease cohorts (coefficients of variation: 9 % and 5 % in AD, 6 % and 2 % in CRL, before and after filtering <50 nm respectively).

Notably, we found that the simple number of aggregates is not necessarily sufficient to discern between disease and control samples. The groups’ differentiation was aided by additional features which can be read out from standard MAPTau (brightness ≈ size) and MAPTau extended via super‐resolution or co‐localization analyses (size and composition). Tau aggregates with a wide variation in size and shape are formed in both healthy and AD brains. However, changes in the morphology of sub‐populations of tau aggregates appear to be an important indicator of disease pathology and AD progression.

### Aggregate Composition is Accessible via Co‐Labelling of Multiple Epitopes

High heterogeneity in the frequency and occurrence of cerebral tau phosphorylation has been observed between AD patients.[^35^, ^36^] While certain sites (mostly towards the N and C termini, rather than the center) are phosphorylated only in disease state, phosphorylation of other sites also occurs in healthy adult human brains, although with much lower prevalence compared to AD.^[35]^ Meanwhile, sites, such as T231, show a progressive increase in phosphorylation with disease state.^[37]^ Importantly, an overall increase in tau phosphorylation as well as changes in tau phosphorylation sites have been linked to progression in AD.^[38]^ However, studies so far have focused on total tau (not aggregate specific) and used methods unable to distinguish individual molecules (ELISA, mass spectrometry), giving an average of the phosphorylation sites of the entire aggregate population. While these techniques can identify the presence of different modifications in tau aggregates, they are not capable of detecting whether these modifications co‐occur in the same aggregates. Thus, little is known about the phosphorylation status of individual aggregates and a potentially causal relationship between certain modifications.

**Figure 4:**
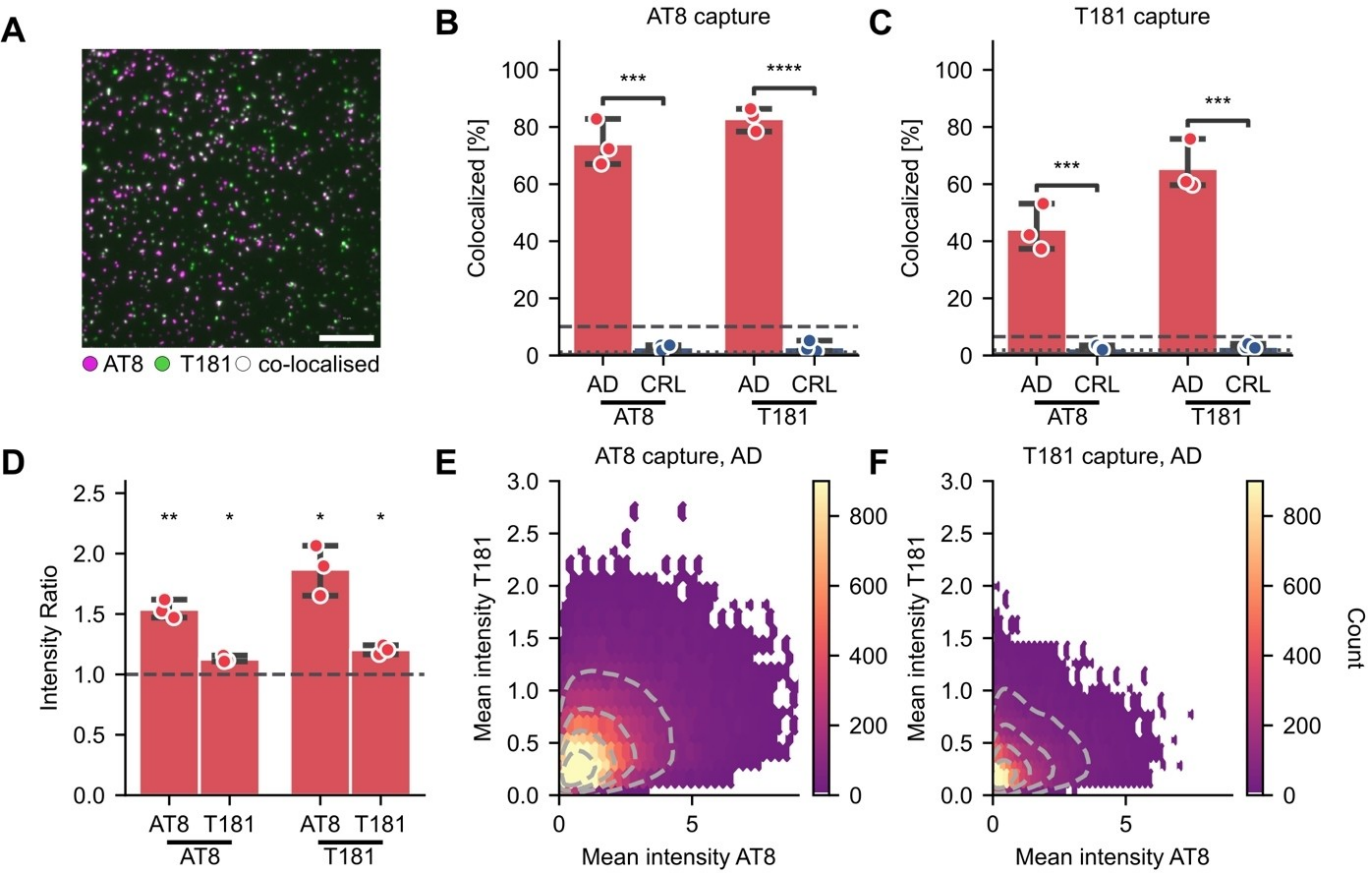
Co‐localization of antibodies targeting tau phosphorylation sites (T181, AT8) reveals differences in disease‐associated aggregate composition. (A) Representative image of co‐localized spots in AD brain homogenate. Scale bar=10 μm. (B–C) Percentage of green spots (T181) co‐localized with magenta spots (AT8) and vice versa using (B) AT8 or (C) T181 to capture aggregates from brain homogenate. (D) Ratio of the brightness of co‐localized and non‐colocalized spots for each channel in B, C. (AT8: 638 nm, T181: 488 nm) detected in AD samples using either AT8 or T181 for capture. (E–F) Mean intensity of each co‐localized spot in AD samples using (E) AT8 or (F) T181 to capture. Panel B and C show the mean±S.D. of *n*=3 biological replicates in each disease cohort, compared by t‐test. *: *p*<0.05, **: *p*<0.01, ***: *p*<0.001. Panel D: one‐sample t‐test against a hypothetical value of 1 (equivalent to no difference between the co‐localized and non‐colocalized spots).

We next sought to characterize the composition of tau aggregates by adapting MAPTau for two‐color coincidence detection. This enabled us to detect several phosphorylation sites present in the same aggregate simultaneously, namely p‐Ser202 and p‐Thr205 (detected by AT8), and p‐Thr181 (detected by T181), both centrally located in the protein. We captured tau aggregates either with AT8 or T181 antibodies and then detected them with a mixture of both the AT8 and T181 antibodies labelled with spectrally distinct fluorophores (Alexa Fluor 647 and 488, respectively, Figure 4A). By detecting the two‐color coincidence, these configurations ensure that we detect aggregates (i.e., particles containing two copies of the capture antibody epitope) decorated with the modifications of interest.

To quantify co‐localization, we detected diffraction‐limited spots labelled by one fluorophore and then calculated the proportion of those spots that were also labelled with the second fluorophore. We detect phosphorylation of both sites in AD and control brain samples with elevated levels in AD. Interestingly, almost no co‐localization of the T181 and AT8 antibodies was observed in control brain samples (no more than would occur by chance; Figure 4B dotted line, obtained from rotating one channel relative to the other). However, in the AD sample more than 75 % of T181‐positive tau aggregates were also positive for AT8, and vice versa (Figure 4B), significantly higher than in the control samples (t‐test: AT8 detection p=0.00011, T181 detection p=0.0000066). Comparable observations were made using T181 to capture tau aggregates (Figure 4C, t‐test: AT8 detection p=0.00090, T181 detection p=0.00028). Finally, we combined the co‐localization and brightness analyses. We found that in both detection colors co‐labelled aggregates were significantly brighter than singly labelled aggregates in either antibody configuration (Figure 4D, one‐sample t‐test, AT8‐AT8: p=0.0065, AT8‐T181: p=0.014, T181‐T181: p=0.011, T181‐AT8: p=0.018). Indeed, co‐labelled aggregates were up to twice as bright (~large) in comparison to singly labelled aggregates in the AD brain samples. These studies could be expanded in the future by determining the size of the co‐localized aggregates to assess whether the aggregates are indeed bigger (i.e., contain more total tau) or the proportion of phosphorylated sites is increased. Lastly, we observed a significant positive correlation (AT8 capture: Pearson's coefficient=0.36, T181 capture: Pearson's coefficient=0.28) in the brightness of individual aggregates between channels indicating that, despite being heterogeneous across the population, aggregates with more AT8 phosphorylation sites also tend to have increased T181 phosphorylation (Figure 4E and F). We anticipate this method will be readily applicable to other tau aggregate species from various biological samples using antibodies targeting a range of characteristics including post‐translational modifications, conformation, and isoforms.[^39^, ^40^, ^41^] Overall, these data demonstrate that MAPTau is amenable to compositional studies of tau aggregates that reveal additional discriminators between AD and control cohorts.

Measuring the composition of the individual aggregates, i.e., the occurrence of several phosphorylation sites in a single aggregate, is not readily possible with bulk techniques. While other single‐molecule methods to study tau aggregates exist, which report a digital assay readout of diffraction‐limited pixel positivity, it is not amenable to characterizing aggregate size or shape.^[42]^ The results from our study indicate that those features might play a greater role in disease pathology compared to aggregate abundance alone, as we found significantly more large tau aggregates in AD brains.

### MAPTau Detects Tau Aggregates in Serum

**Figure 5:**
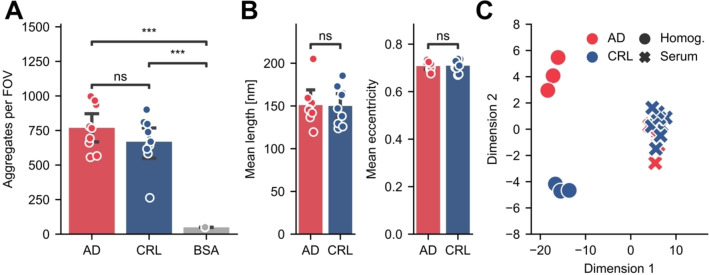
Quantification of total tau aggregates in human serum. (A) MAPTau quantification of HT7‐positive aggregates in human serum samples from AD and control donors, and a BSA negative control. (B) Mean length and eccentricity in serum tau aggregates determined by super‐resolution microscopy. (C) Linear discriminant analysis (LDA) of human serum (*n*=9) and brain homogenate (*n*=3) samples from AD and control patients. Panel A and B show the mean±S.D. of *n*=9 biological replicates (BSA *n*=2 technical replicates). Panel A reports a one‐way Anova with post‐hoc Tukey HSD test, Panel B reports Student's t‐tests. ns: *p*>0.05, **: *p*<0.01, ***: *p*<0.001.

We confirmed our assay is compatible with readily available and clinically relevant samples, such as serum, increasing the utility of MAPTau, as it can be used for disease diagnosis while the patient is still alive.^[43]^ For this purpose, we tested 9 human serum samples obtained from AD patients and 9 from healthy control patients. We detected significantly more HT7‐positive spots in human serum samples compared to a tau‐negative BSA control (Figure 5A, one‐way Anova: *p*=0.00020; post‐hoc Tukey HSD test, *p*=0.00064 for CRL vs BSA, *p*=0.00013 for AD vs BSA), confirming the compatibility of MAPTau with human serum. We were able to detect an average of 770±160 HT7‐positive spots per FOV in the AD serum and 670±170 in the control serum. However, similar to the brain, there was no significant difference in the number of spots in serum from AD versus control patients (*p*=0.44). We did not observe any correlation to the age and sex of the donors. We further confirmed that MAPTau is capable of super‐resolving tau aggregates in serum samples, characterizing the morphology of the serum‐derived aggregates. We did not detect significant differences in either length or eccentricity between the HT7‐positive tau aggregate population in AD compared to control samples (Figure 5B). The successful application of MAPTau in human serum shows its compatibility with clinically relevant biofluids.

### MAPTau Reveals Heterogeneity Among Tau Aggregates Derived from Tissue versus Biofluids

Lastly, we compared serum‐ with brain‐derived tau aggregates across both AD and control cohorts employing a comprehensive analysis approach. Instead of looking at all parameters individually, we reasoned the combination of all morphological information may better encapsulate subtle differences in the aggregate populations both between tissue types and disease cohorts. We compiled the mean morphological parameters collected for each aggregate population, including length, area, perimeter, and eccentricity supplemented with the number of aggregates, number of localizations per aggregate, and major and minor axis length. A pairwise comparison of all the features is shown in Supplementary Figure S3. These parameters were used for linear discriminant analysis (LDA), a method commonly used for supervised dimensionality reduction which maximizes the separation between different categories using linear combinations of the provided parameters.

Using this method, it was possible to examine whether the HT7‐positive aggregates detected in serum differed from those observed in human brain homogenate, as well as any differences between AD and control cohorts. Thus, the dataset included four different aggregate populations; namely, HT7‐positive aggregates from control or AD serum, and from control or AD brain homogenate. Interestingly, when projected onto a two‐dimensional parameter space, we observed the sample cohorts formed three clusters driven primarily by eccentricity, minor and major axis length (Figure 5C). Brain homogenate and serum samples (AD brain vs control brain vs AD serum vs control serum) were well separated by this method, clustering to opposite sides of the first dimension. This indicates that the tau aggregates in serum are distinguishable from the brain‐derived aggregates according to the parameters collected from the HT7 MAPTau assay. In addition, AD and control brain‐derived samples formed two distinct clusters separated in the second dimension, indicating aggregates from these samples also have distinguishing morphological features captured by the HT7 MAPTau analysis. However, no separation between the aggregates from AD and control serum was observed in this dimension. This is consistent with recent studies which showed that tau found in blood mostly originates from peripheral tissues and is not brain derived,^[44]^ resulting in a lack of correlation between serum and CSF total tau.^[45]^ If the majority of tau aggregates in serum are also composed of non‐brain‐derived tau, it is perhaps unsurprising that we do not detect differences in this population associated with neurodegenerative disease.

## Conclusion

We describe here MAPTau, a new assay that enables the detection and characterization of tau aggregates in a variety of biological samples with high specificity and sensitivity, using various single‐aggregate characteristics. This assay measures aggregate number, morphology, and composition. This technology makes temporal monitoring of such systems feasible, producing longitudinal data with which it would be possible to tackle fundamental mechanistic questions such as the order of phosphorylation before and after aggregate formation, or whether phosphorylation at particular sites triggers hyperphosphorylation. MAPTau is sufficiently sensitive to detect small soluble tau aggregates which cannot be readily detected by many of the established techniques. Thus, it may be possible to use MAPTau to monitor tau pathology as it progresses with disease. Overall, the specificity and sensitivity of MAPTau coupled with its ease of use and low sample volume requirements (<10 μL) make it perfectly suited to characterize model systems or clinical samples.

## Supporting Information

Postmortem brain tissue from three donors with AD and three age‐matched control donors was acquired from the Cambridge Brain Bank (with the approval of the London–Bloomsbury Research Ethics Committee; 16/LO/0508, Supporting Information Table S1; and with written informed consent from either the donor ante mortem or from their next of kin postmortem in accordance with UK law). The brain samples were voluntarily donated without any compensation. Serum samples from ten people with amnestic Alzheimer's disease (with dementia or mild cognitive impairment supported by imaging and/or biomarker evidence of AD) and ten controls were provided ante mortem after written informed consent from volunteers with mental capacity (with the approval of East of England Cambridge Central Research Ethics Committee 15/EE/0270; see Supporting Information Table S2).

## Conflict of interests

The authors declare no conflict of interest.

1

## Supporting information

As a service to our authors and readers, this journal provides supporting information supplied by the authors. Such materials are peer reviewed and may be re‐organized for online delivery, but are not copy‐edited or typeset. Technical support issues arising from supporting information (other than missing files) should be addressed to the authors.

Supporting Information

## Data Availability

Exemplar images for diffraction‐limited and super‐resolved analyses have been provided alongside summaries of the pre‐processed quantitative data via Zenodo DOI: 10.5281/zenodo.8020036. Any other data supporting this study are available from the corresponding author upon reasonable request.
