# Supplementary material for: Single‐Molecule Characterization and Super‐Resolution Imaging of Alzheimer's Disease‐Relevant Tau Aggregates in Human Samples
Source: Angew Chem Int Ed Engl. 2024 Apr 17;63(21):e202317756. doi: 10.1002/anie.202317756 (PMC11497306; doi:10.1002/anie.202317756)
Supplement: Supplementary file 1 — Supporting Information [file ANIE-63-e202317756-s001.pdf]

Angewandte Chemie
Eine Zeitschrift der Gesellschaft Deutscher Chemiker

Supporting Information

Single-Molecule Characterization and Super-Resolution Imaging of Alzheimer's Disease-Relevant Tau Aggregates in Human Samples

*D. Böken, D. Cox, M. Burke, J. Y. L. Lam, T. Katsinelos, J. S. H. Danial, E. Fertan, W. A. McEwan, J. B. Rowe, D. Klenerman**

Supplementary Materials for

Single-Molecule Characterization and Super-Resolution Imaging of Disease-Relevant Tau Aggregates in Human Samples

Dorothea Böken *et al.*

*Corresponding author. Email: dk10012@cam.ac.uk

This PDF file includes:

Supplementary information
Supplementary Figs. 1 to 5
Supplementary Tables 1 to 2

Methods

Preparation of recombinant aggregates

Lyophilized monomeric recombinant Aβ42 peptide (Stratech, Cat. No. A-1170-2-RPE-1.0mg) was dissolved in PBS (pH = 7.4) at 200 μM on ice. The solution was quickly aliquoted and snap frozen. To prepare recombinant Aβ42 fibrils, an aliquot was thawed and diluted to 4 μM in 1xPBS supplemented with 0.01% NaN3 (Merck, Cat. No. 71290) and incubated at 37 °C under quiescent conditions for one week. The Aβ42 fibril was then sonicated as described previously[46] with modification. The one-week aggregated Aβ42 aliquot was immersion sonicated in an ice water bath with a 3-mm-titanium probe (Sonicator microprobe 4422, Qsonica) mounted on a tip sonicator (Ultrasonic processor Q125, QSonica) at 20 kHz with 40% of power for 24x5-s bursts with 15-s rests between bursts. Thereafter, the sonicated aggregate was centrifuged, aliquoted (50 μL) and snap frozen. The aliquots were stored at -80 °C until use.

Wild type α-synuclein was expressed, purified in E. coli and stored at -80 °C as described previously[47]. To remove pre-aggregation seeds, the solution was ultracentrifuged at 91,000 g at 4 °C for 1 hour (Optima TLX Ultracentrifuge, Beckman). The concentration of the supernatant was then determined by A280 (ε280 = 5,960 M−1 cm−1). The supernatant was then diluted to 70 μM in 1xPBS supplemented with 0.01% NaN3 and incubated at 37 °C with shaking at 200 rpm for two months.

Recombinant N-terminally 6xHis-tagged human P301S 0N4R tau was expressed and purified from E. coli BL-21 DE3 cells. Protein expression was induced by addition of 0.5 mM IPTG at 16 °C overnight. Cells were pelleted (17,000 x g, 3 min) and lysed in recombinant tau-lysis buffer (1 mM benzamidine, 1 mM PMSF, 1x cOmplete™, EDTA-free Protease Inhibitor Cocktail mix (Merck), 14 mM b-mercaptoethanol, 300 mM NaCl, 25 mM HEPES, 30 mM imidazole, 1% NP-40). Purification was performed on the AKTA Pure using the HisTrap HP column (Cytiva), followed by size exclusion chromatography using a Superdex 200 HiLoad 16/600 pg column as previously described (PMID: 29768203). Tau monomer fractions were stored in 1x PBS buffer, freshly supplemented with 1 mM DTT. In vitro aggregated tau assemblies were prepared by addition of heparin at 37 °C for 3 days while shaking, using monomeric tau at 60 μM in the presence of 20 μM heparin (Sigma Aldrich) in PBS supplemented with 2mM DTT and 1x cOmplete™, EDTA-free Protease Inhibitor Cocktail mix. A small aliquot of the assemblies was kept for analysis and the remaining material was sonicated for 15 sec before long-term storage at -80 °C.

The HT7 epitope-containing peptides were designed with the following sequences GAA**PPGQK**GQ{PEG4}GAA**PPGQK**GQ to mimic dimeric particles and GAA**PPGQK**GQ{PEG4}GKPQPAGAQG to mimic monomeric particles, where PPGQK corresponds to the immunogen's amino acid sequence (HT7: abcam, Cat. No. MN1000). These peptides were synthesized by GenScript and provided as lyophilized powder before being resuspended in milliQ water at a final concentration of 2 mg/mL. Stocks were stored at -20 °C, then diluted to the desired concentration in PBS immediately before use.

Isolation of cell-derived tau aggregates

HEK293 cells expressing tau P301S-Venus (PMID: 28049840) were maintained in DMEM supplemented with 10% FCS, 100 U/ml penicillin, 100 ug/ml streptomycin and grown at 37 °C and 5% CO2. The cells were seeded with 50 nM heparin-assembled recombinant 6xHis-tau P301S assemblies in the presence of 1% Lipofectamine2000 and by serial dilutions, a single clone was isolated (R1E5) that stably propagates tau P301S-Venus aggregates. Cells were lysed in R1E5-Lysis buffer (1x PBS, 1% w/v Triton X-100, 1x cOmplete™, EDTA-free Protease Inhibitor Cocktail mix, 1x PhosSTOP™ phosphatase inhibitor mix) on ice for 30 min. The lysate was subsequently centrifuged at 14, 000 × g for 15 min at 4 °C and the clarified lysate was aliquoted and stored at -20 °C.

Homogenization of Brain Samples

Brains were flash-frozen and stored at −80 °C at the Cambridge Brain Bank in Cambridge. Fresh-frozen brain tissue was homogenized using a method adapted from Goedert, et al.[48]. Briefly, the tissue was homogenized at 4 °C in a VelociRuptor V2 Microtube Homogenizer (Scientific Laboratory Supplies, Cat. No. SLS1401) in 10 volumes of homogenization buffer (10 mM Tris-HCl, 0.8 M NaCl, 1 mM EGTA, 0.1% Sarkosyl, 10% sucrose; pH 7.32) containing cOmplete™ Ultra Protease Inhibitor and PhosStop™ Phosphatase Inhibitor. The homogenate was centrifuged at 21,000 × g for 20 minutes at 4 °C, and the upper 90% of the supernatant was retained. The pellet was re-homogenized in 5 volumes of homogenization buffer, then centrifuged at 21,000 × g for 20 minutes at 4 °C. The upper 90% of this supernatant was then removed and combined with the first supernatant, and this mixture was aliquoted and frozen at -80 °C until used for further experiments. Total protein concentration was determined using a BCA assay (Thermo Fisher, Cat. No. 23227) as per the manufacturer's instructions. The concentration of tau was determined using the Human Tau ELISA kit (abcam, Cat. No. ab273617) according to the manufacturer's instructions.

Coverslip passivation

Coverslip passivation for SiMPull was completed using a method adapted from Chandradoss, et al.[49]. 26 mM × 76 mM #1.5 borosilicate glass coverslips (VWR, Cat. No. MENZBC026076AC40) were washed in a sonication bath for 10 minutes each in 18.2-MΩ·cm water, acetone, and methanol, respectively, then 20 minutes in 1 M KOH. Coverslips were rinsed with 18.2-MΩ·cm water and then methanol, then dried with nitrogen, then cleaned with argon plasma for 15 minutes (Femto Plasma Cleaner; Diener Electronic, Royal Oak, MI, USA). Next, coverslips were silanized in a 3:5:100 mixture of (3-Aminopropyl)triethoxysilane (APTES) (Fisher Scientific UK, Cat. No. 10677502), acetic acid, and methanol, respectively. Coverslips were sonicated in this solution for 1 minute, left undisturbed for 10 minutes, sonicated again for 1

minute, then left undisturbed for an additional 10 minutes. Coverslips were then rinsed twice with 18.2-MΩ·cm water and once with methanol, then dried with nitrogen. One 50-well silicone gasket (Grace Bio-Labs, SKU 103250) was then attached to the surface of each coverslip. Each well was passivated by firstly introducing 9 µL of a freshly prepared 100:1 aqueous mixture of SVA-PEG-OMe (110 mg/mL, Mw ~5,000; Laysan Bio Inc., Cat. No. MPEG-SVA-5000) and SVA-PEG-Biotin (100 mg/mL, Mw ~5,000; Laysan Bio Inc., Cat. No. Biotin-PEG-SVA-5000) followed by adding 1 µL of 1 M NaHCO3 (pH 8.5). Coverslips were left covered in a humidity chamber for 24 hours, then rinsed twice with 18.2-MΩ·cm water, then dried with a nitrogen stream. Each well was then further passivated by adding 9 µL of a freshly prepared aqueous solution of methyl-PEG4-NHS-Ester (10 mg/mL, Thermo Fisher, Cat. No. 22341), followed by adding 1 µL of 1 M NaHCO3 (pH 8.5). Coverslips were left covered in a humidity chamber for 24 hours, then rinsed twice 18.2-MΩ·cm water, dried with nitrogen, and then stored desiccated at -20 °C until used for experiments.

Antibody Labelling

Biotin-conjugated AT8 and HT7 are commercially available (AT8: Invitrogen, Cat. No. MN1020B; HT7: Invitrogen, Cat. No. MN1000B). T181 (abcam, Cat. No. ab232849 and SC211 (Santa Cruz, Cat. No. sc-12767L) were biotinylated site-specifically through Click chemistry, using the SiteClick Antibody Azido Modification kit (Invitrogen, Cat. No. S20026). Briefly, 200 µg of the antibody was buffer-exchanged and concentrated to ~2 mg/ml and subsequently incubated with β-galactosidase overnight. The antibody was then incubated overnight in the presence of β-1,4-galactosyltransferase to attach azide modified carbohydrates (UDP-GalNAz) to the modified glycan chains. Subsequently, the antibody was purified using a 50 kDa Amicon Ultra spin column (Merck). Ten equivalents of DBCO-PEG4-biotin (Sigma Aldrich, Cat. No. 760749) were added to the antibody and incubated overnight at 37 °C. The biotin-labelled antibody was purified using a 50 kDa Amicon Ultra spin column, and the concentration was quantified by A280. Antibodies were fluorescently labelled with AF647 or AF488 using the Alexa Fluor® Conjugation Kit (Fast) - Lightning-Link (Abcam, Cat. No. ab269823 and ab236553) according to manufacturer's instructions. Excess fluorophore was removed using a 50 kDa Amicon Ultra Spin column (Merck) and a 40 kDa Zeba Spin desalting column.

Single-molecule Pulldown (SiMPull)

SiMPull for tau aggregates was adapted from[50]. Briefly, wells were washed once with PBST (PBS (50 mM tris base and 150 mM NaCl, pH 7.4) containing 0.05% Tween-20). 10 µL NeutrAvidin (0.2 mg/mL in PBST; Thermo Fisher, Cat. No. 31000) was added to each well and left to incubate for 10 minutes. After a washing sequence (two 10-µL washes of PBST, followed by one 10-µL wash of PBS containing 1% Tween-20), biotinylated capture antibodies (AT8: Invitrogen, REF MN1020B; HT7: Invitrogen, REF MN1000B; T181: ab232849 were diluted to 10 nM in blocking solution (1 mg/mL BSA in PBST), and left to incubate for 10 minutes. After a washing sequence, wells were blocked with blocking solution for 10 minutes. After one PBST wash, 10 µL of sample was added to each well and left to incubate. Brain homogenate samples were diluted 1:10 in PBS and incubated for 1h at room temperature, serum samples were diluted 1:2 in PBS and incubated overnight at 4 °C, followed by a washing sequence. Fluorescently labelled detection antibodies (AT8: Invitrogen, Cat. No. MN1020, 5 nM; HT7: Invitrogen, Cat. No. MN1000, 2 nM; T181: abcam, Cat. No. ab232849, 5 nM) were added in blocking solution, and left for 15 minutes, followed by a final washing sequence. Wells were then washed once with PBS, then the wells were filled with fresh PBS before the gasket was sealed with another clean coverslip.

Cross-reactivity of tau SiMPull assay

AT8 and HT7 SiMPull assays were performed as described above, using recombinant α-synuclein and amyloid-β aggregates at 1 µM. As a control, SiMPull was performed using amyloid-β (6E10, BioLegend, Cat. No. 803007 and 803021) and α-synuclein (SC211, Santa Cruz, Cat. No. sc-12767) specific antibodies for matched capture and detection.

Diffraction-limited Imaging and Analysis

Imaging was done on a home-built total internal reflection fluorescence (TIRF) microscope, consisting of an inverted Ti-2 Eclipse microscope body (Nikon) fitted with a 1.49 N.A., 60x TIRF objective (Apo TIRF, Nikon) and a perfect focus system. Images were acquired using a 638 nm laser (Cobolt 06-MLD-638, HÜBNER) or 488 nm laser (Cobolt 06-MLD-488, HÜBNER). Detection antibodies labelled with AF647 or AF488 were excited at 638 nm and 488 nm respectively, with the resultant fluorescence collected by the objective, passed through a quad-band dichroic beam splitter and cleaned up using the appropriate emission filter (for 488-nm-induced fluorescence: BLP01-488R-25x36, Semrock and FF01-520/44-25x36, Semrock; for 638-nm-induced fluorescence BLP01-635R-25x36, Semrock).

EMCCD camera (Evolve 512, Photometrics) operating in frame-transfer mode (electron-multiplying gain of 6.3 electrons/ADU and 250 ADU/photon). Each pixel corresponds to a length of 107 nm on the recorded image.

Typically, 16 fields of view (FOVs) of 54.784 µm2 each were imaged per well, each for 50 frames of 50 ms exposure. Each pixel corresponds to a length of 107 nm. Images were collected in a grid using an automated script (Micro-Manager[51]) to avoid any bias in the selection of FOVs. For co-localization experiments, images were acquired sequentially in each excitation channel.

Individual fluorescent spots were quantified using a python-based adaptation of ComDet[52], a package originally developed to identify bright intensity spots in images with a heterogeneous background. Briefly, a mean intensity projection was prepared from the last 40 frames of each field of view to minimize background fluctuations. Thresholds were then optimized for each experiment by comparing the number of particles identified by ComDet in positive and negative control images, such that the threshold was set to the lowest value at which the negative control was less than 1% of the positive

control. This threshold was then used along with a particle size estimate of 4 to identify fluorescent spots. The mean intensity of each spot was measured using scikit-image, and background corrected by subtracting the median intensity of all non-spot pixels for each FOV.

For co-localization experiments, ComDet identification was performed independently on both detection channels and the resultant list of centroid coordinates collected. For each channel, spots co-labelled in the opposing channel were determined as those within a 4-pixel radius according to the Euclidean distance (Supplementary Fig. 4A). In the case where more than one spot met this criterion, the closest spot was selected as the co-localized pair. The chance co-localization was estimated by transposing the centroid coordinates for the second channel in the x-dimension and repeating the threshold distance analysis (Supplementary Fig. 4B). Finally, the proportion of co-localized spots was calculated for a given channel as the number of spots matched to a corresponding spot divided by the total number of spots detected in that channel (Supplementary Fig. 4C).

Super-resolution Imaging and Analysis

STORM buffer was freshly prepared at 1 mg/mL glucose oxidase, 52 µg/mL catalase and 50 mM MEA (cysteamine) in 50 mM Tris in PBS + 10% Glucose, pH 8, and filtered through a 0.02 µm filter. In preparation for STORM imaging, PBS was removed from each well and replaced with STORM buffer before sealing the gasket with another clean coverslip. Imaging was performed with Typically, at least 6000 frames of 33 ms exposure were recorded per field of view.

Super-resolution images were reconstructed using the Picasso[53] package. Briefly, after discarding the first 300 frames, localizations were identified and fit then corrected for microscope drift using the inbuilt implementation of redundant cross-correlation. Localizations were then filtered for precision <30 nm, with a final mean precision of » 12 nm. Random localizations were removed using DBSCAN as provided by the scitkit-learn package[54] with permissive parameters (radius of 2 and minimum density of 1). The resultant clustered array of localizations was then subjected to a series of morphological dilation, closing and erosion operations as provided by the scikit-image package[55] to yield single connected regions of interest corresponding to individual aggregates (Supplementary Fig. 5 A - C). Aggregates with <3 localizations per cluster were considered noise and removed. Each aggregate was then measured using a combination of scikit-image[55] for basic region properties such as perimeter, area, and eccentricity, and SKAN[56] for skeletonized length (Supplementary Fig. 5D) whereby the length of each aggregate is reported as the summed branch distance. Finally, super-resolved images were rendered using the inbuilt Picasso[53] functionality.

Limit of Detection

The limit of detection (LOD) was determined by a serial dilution of a brain homogenate sample obtained from an AD donor. The absolute concentration of tau was determined using the commercially available human tau ELISA. It should be noted that the ELISA was not aggregate specific, thus it reflects the total concentration of all tau (including monomer) in the sample. SiMPull were performed and the resultant particle counts at each homogenate concentration were fit using four-parameter logistic regression (Supplementary Fig. 1C).

The limit of blank (LOB) is the highest apparent number of spots expected to be found when replicates of a sample containing no tau aggregates are detected, and is defined as[26]:

$$LOB = \text{mean blank} + 1.645 \times (\text{SD of blank}) \quad (1)$$

The limit of detection (LOD) was determined by utilizing the previously determined LOB and replicates of a sample containing a low concentration of tau aggregates, and is given by the expression[26]:

$$LOD = LOB + 1.645 \times (\text{SD of low concentration sample}) \quad (2)$$

The LOB for the HT7 assay was determined as 575 pg/mL and the LOD as 874 pg/mL. The LOB for the AT8 assay was determined as 591 pg/mL and the LOD as 2201 pg/mL.

Statistical analysis

Statistical analyses were performed using either the SciPy[57] or statsannotations[58] packages in python. The exact p values and statistical details are provided in the main text or figure legends as appropriate.

Data availability

Where feasible, exemplar images for diffraction-limited and super-resolved analyses have been provided alongside summaries of the pre-processed quantitative data via Zenodo DOI: 10.5281/zenodo.8020036. Any other data supporting this study are available from the corresponding author upon reasonable request.

Code availability

Analyses presented in this manuscript rely on various published python packages including Picasso[53], scikit-learn[54], scikit-image[55], and SKAN[56]. All other custom python scripts used in this study are available via Zenodo DOI: 10.5281/zenodo.8027256.

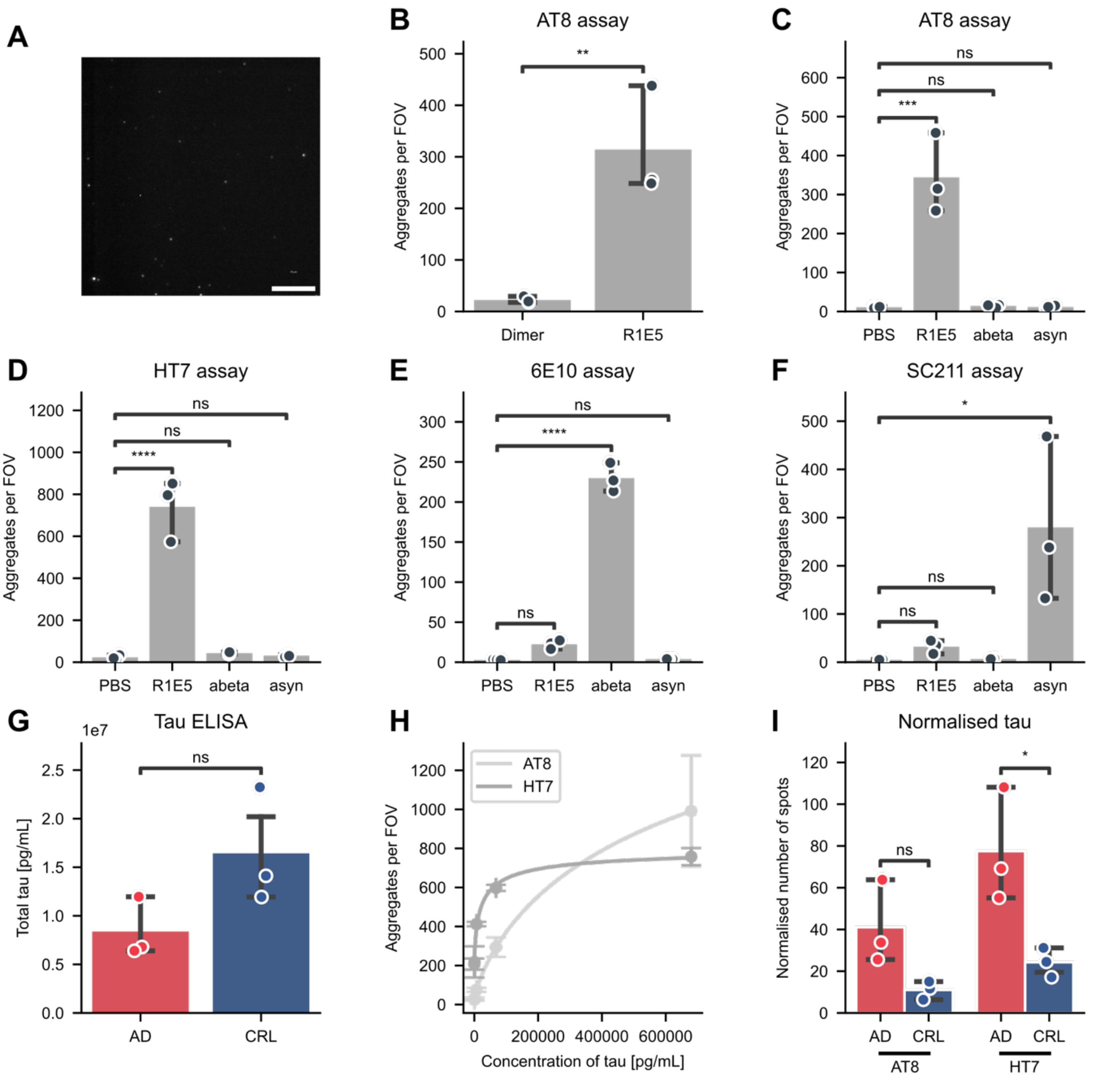

Supplementary Figure 1. Validation of the tau SiMPull assay. (A) Representative image of a sample containing no tau (BSA) where no fluorescent spots are quantified. (B) Quantification of fluorescent spots observed using the HT7-epitope dimer-mimicking peptide in an AT8 tau SiMPull assay. As a positive control, a cell lysate from HEK cells overexpressing hyperphosphorylated tau aggregates (R1E5) was used. (C-D) SiMPull assays against (C) AT8 or (D) HT7 were tested for cross-reactivity with recombinant amyloid-β and α-synuclein aggregates. (E-F) SiMPull assays against (E) amyloid-β and (F) α-synuclein demonstrate recombinant aggregates of amyloid-β and α-synuclein can be successfully detected by their respective SiMPull assays. (G) Total tau concentration in AD and control brain homogenate samples determined using a commercial tau ELISA kit (not aggregate specific). (H) Four-parameter logistic regression fitted to the number of aggregates per FOV for a serial dilution of a highly concentrated tau aggregate sample (Braak VI brain homogenate). (I) Tau aggregate levels normalized to the total tau levels determined through ELISA. Panel B - H show the mean ± S.D. of n=3 technical replicates compared using a t-Test (panel B, G) or a one-way Anova with post-hoc Tukey HSD test (panel C-F). ns: $p > 0.05$, *: $p < 0.05$, **: $p < 0.01$, ***: $p < 0.001$, ****: $p < 0.0001$.

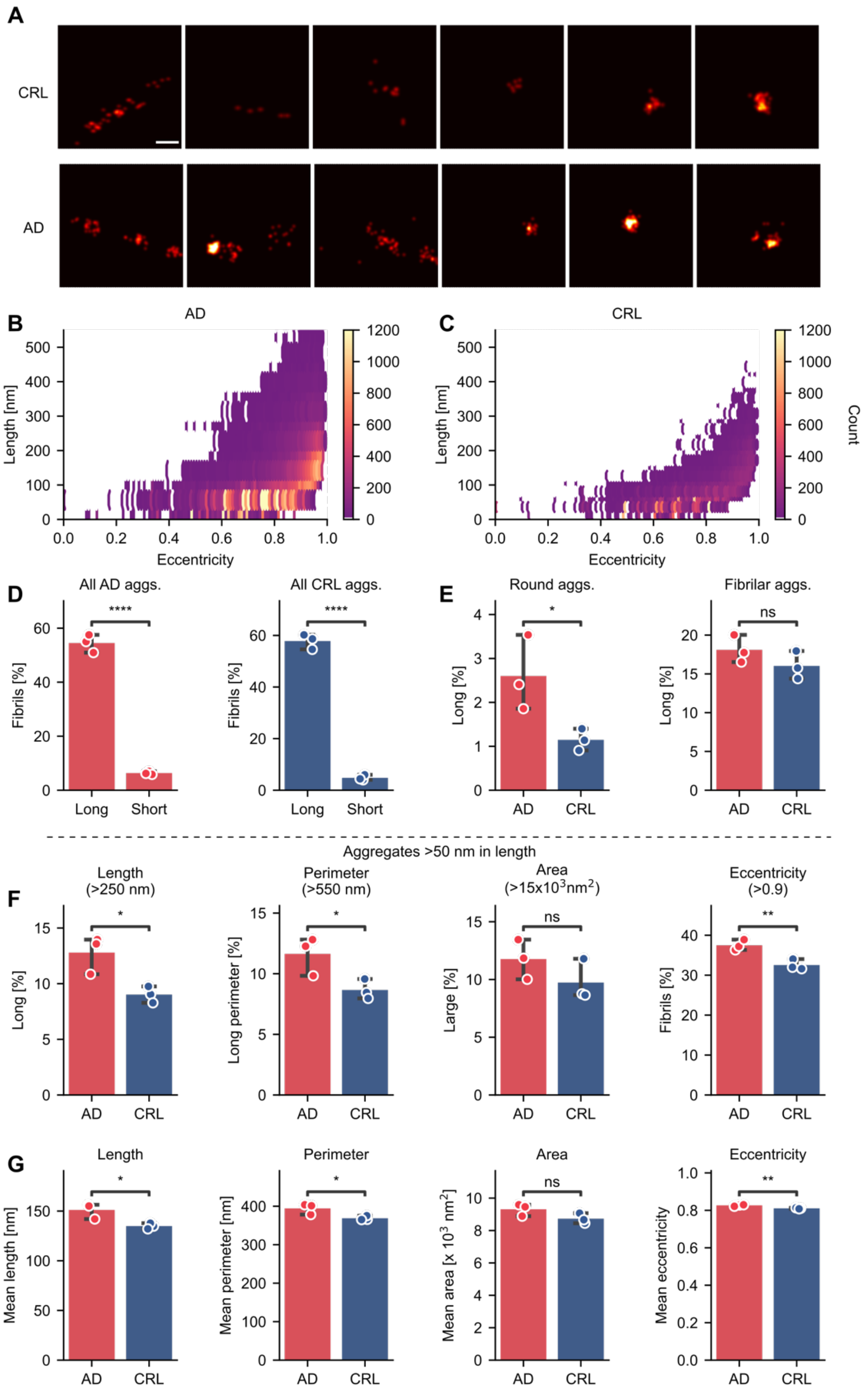

Supplementary Figure 2. Morphological characterization of super-resolved tau aggregates derived from human brain homogenate. (A) Representative images of aggregates observed in human brain homogenate from AD and control patients, showing fibrillar aggregates and smaller, round aggregates. Scale bar = 100 nm. (B) Length and eccentricity of the individual aggregates in AD brain homogenate samples. (C) Length and eccentricity of the individual aggregates in control brain homogenate samples. (D) Percentage of fibrils (eccentricity >0.9) in aggregates classified as either long (>250 nm) or short (<100 nm) in AD (red) and control brain (blue) homogenate. (E) Percentage of long aggregates (>250 nm) in aggregates classified as round (eccentricity <0.7) or fibrillar (eccentricity >0.9) aggregates in AD (red) and CRL (blue) brain homogenate. (F) Percentage of large and fibrillar aggregates (length: >250 nm, perimeter: > 550 nm, area > 15x 103 nm2, eccentricity > 0.9) after filtering out aggregates <50 nm in length. (G) Mean length, perimeter, area and eccentricity of aggregates after filtering out aggregates <50 nm in length. Panel D - I show the mean ± S.D. of n=3 biological replicates compared using a t-Test. ns: $p > 0.05$, *: $p < 0.05$, **: $p < 0.01$, ****: $p < 0.0001$.

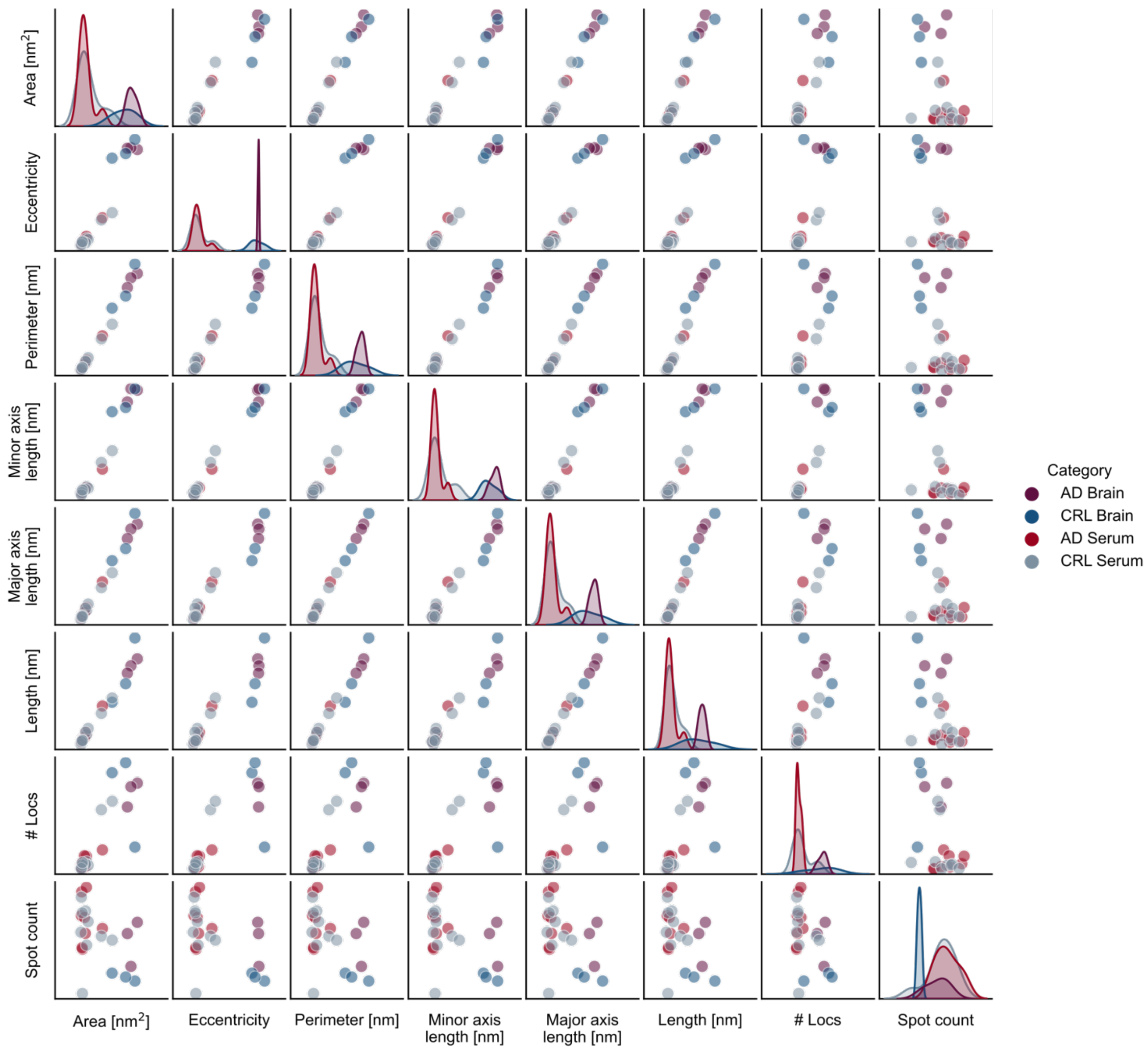

Supplementary Figure 3. Pairwise comparison of morphological features of tau aggregates from brain homogenate and serum. Pairwise relationship of morphological features of tau aggregates (HT7) in human brain homogenate and serum.

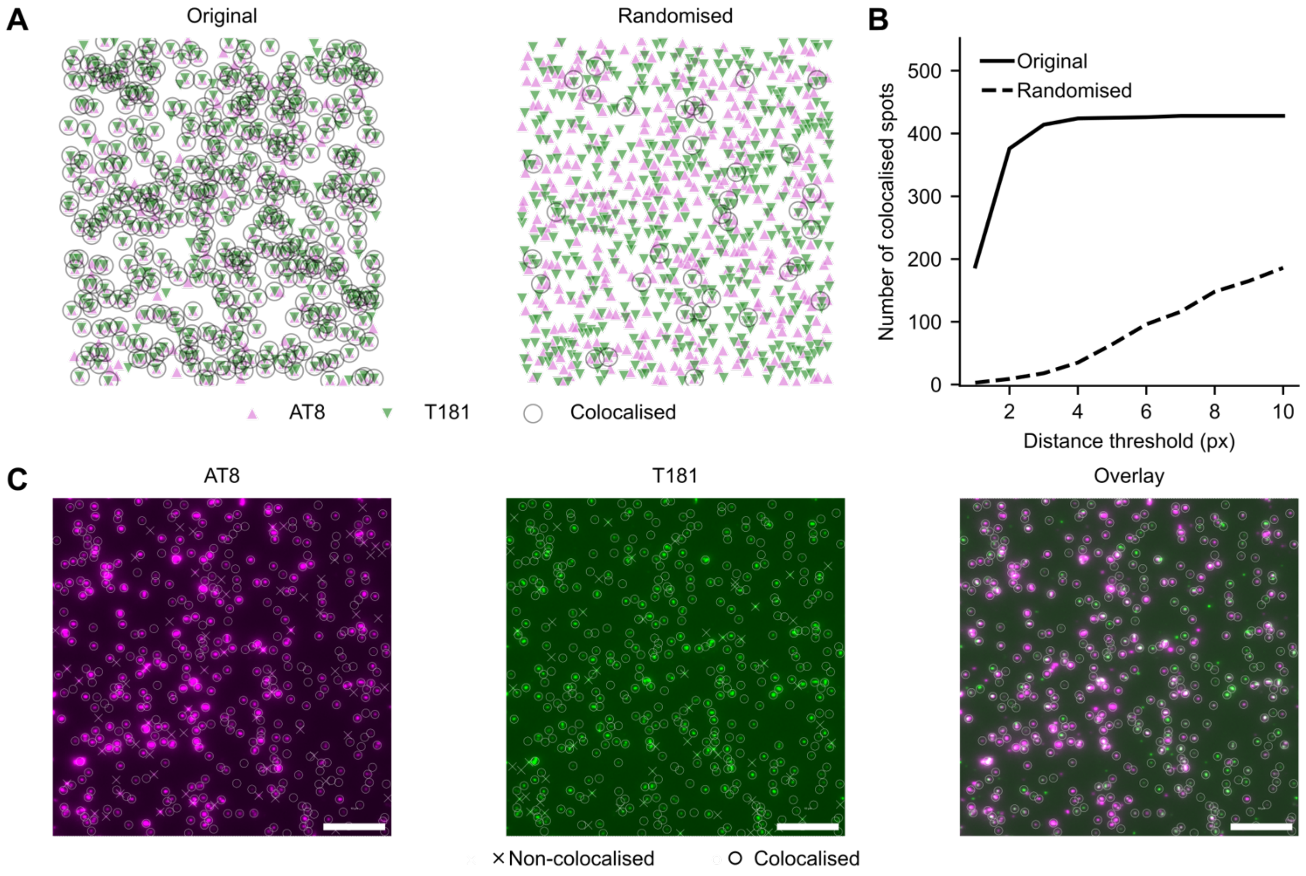

Supplementary Figure 4. Co-localization analysis. (A) Exemplar visualization of spots detected by ComDet in each channel (in this case, AF647-labelled AT8 △ and AF488-labelled T181 ▽). Spots for a given channel are compared with those from the opposing channel, and pairs of spots for which the Euclidean distance is less than the threshold value are considered co-localized (O). In the event that more than one spot passes the threshold, the spot with the shortest distance is selected. To estimate the likelihood of these spots being co-localized by chance, the second channel coordinates are inverted, and the co-localization calculation repeated (Randomized). (B) The number of co-localized spots as calculated for the original or randomized spots shown in A at threshold distances ranging from 1 to 10. (C) Detected spots shown in A overlayed onto the source AT8 or T181 images, demonstrating those which were found to be co-localized (O) or non-colocalized (X). Scale bar = 10 µm.

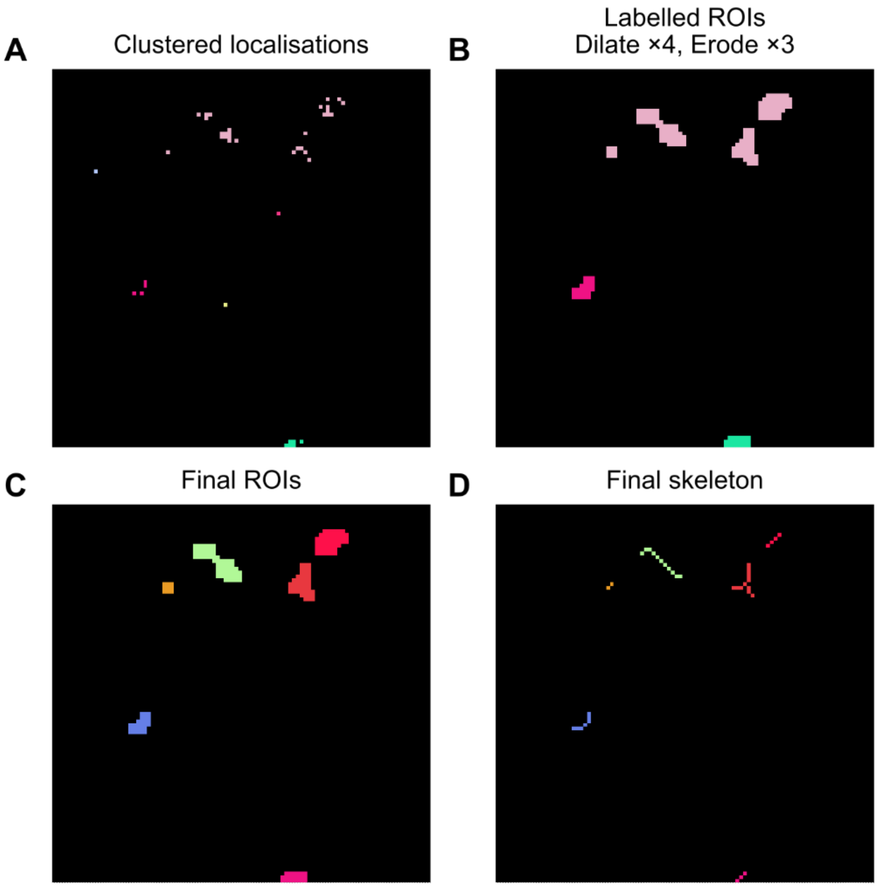

Supplementary Figure 5. Super-resolution measurement of individual molecules. (A) Localizations are first clustered using DBSCAN with permissive parameters to discard isolated localizations (~noise). (B) The resultant localizations are subjected to rounds of morphological dilation, closing and erosion to arrive at single connected regions of interest (ROIs). (C) The resultant ROIs are then relabeled such that individual ROIs are given a single unique identifier. (D) Each ROI is skeletonized to allow for length measurements. In all panels, pixel color represents an arbitrary pixel/object identifier.

Table S1. Brain Homogenate Patient Information.

Subject ID	Sex	Brain region	Group	Age at Diagnosis [years]	Age at Death [years]	Braak Stage
NP16.00028	M	Frontal cortex	Control		68	I
NP18.00159	F	Frontal cortex	Control		68	0
NP19.00009	M	Frontal cortex	Control		66	I
NP14.00055	F	Frontal cortex	AD	64	70	VI
NP17.00246	F	Frontal cortex	AD	67	74	VI
NP18.00042	F	Frontal cortex	AD	86	100	VI

Table S2. Serum Patient Information (CRL = control, AD = Alzheimer's disease).

Group	Sex	Age at visit [years]	Symptom duration [years]	Diagnosis duration [years]
CRL1	M	81.0		
CRL2	F	70.0		
CRL3	F	65.0		
CRL4	F	56.0		
CRL5	F	73.0		
CRL6	M	67.0		
CRL7	M	81.0		
CRL8	M	87.0		
CRL9	M	54.0		
CRL10	M	88.0		
AD1	F	60.3	2.8	2.3
AD2	F	62.3	5.9	2.3
AD3	F	53.8	3.3	1.7
AD4	F	59.8	5.7	3.7
AD5	M	62.3	3.7	2.1
AD6	F	68.9	8.1	1.6
AD7	F	78.1	2.5	0.7
AD8	M	56.5	7.1	1.1
AD9	M	59.6	7.1	2.1
AD10	F	57.5	6.6	2.1
